# Alternative methods of estimating the water potential at turgor loss point in *Acer* genotypes

**DOI:** 10.1186/s13007-019-0410-3

**Published:** 2019-04-04

**Authors:** Jonathan M. Banks, Andrew D. Hirons

**Affiliations:** 1 School of Agriculture Policy and Development, The University of Reading, Reading, Berkshire RG6 6AR UK; 2 Bartlett Tree Experts, Research Laboratory, Reading, Berkshire RG2 9AF UK; 3 University Centre Myerscough, Bilsborrow, Preston, Lancashire PR3 0RY UK

**Keywords:** Turgor loss point, Pressure–volume curve, Drought tolerance, Pressure-bomb, Osmometer, Hygrometer

## Abstract

**Background:**

Selecting for drought tolerance in urban tree species can have a significant influence on survival rates, aftercare requirements and performance. The water potential at turgor loss point (π_tlp_) is gaining popularity as a trait to help determine drought tolerance to aid tree selection. Therefore, it is important to understand if differing methods used to measure or calculate π_tlp_ deliver consistent results.

**Results:**

The sensitivity of three methods used to determine this valuable selection parameter were evaluated. A classical pressure chamber, pressure–volume (P–V) curve method was compared with vapour-pressure osmometer (Vapro^®^) and dewpoint hygrometer (WP4C) methods. These methods were evaluated using closely related cultivars of *Acer platanoides* and *A. pseudoplatanus* ‘Negenia’.

**Conclusion:**

Both the osmometer and hygrometer methods ranked genotypes with a very high similarity (R_s_ = 1, R^2^ = 0.96) and were able to identify significant differences between cultivars. This is the first study to demonstrate suitability of the dewpoint hygrometer in comparison to the vapour-pressure osmometer to measure π_tlp_. The P–V method was unable to identify differences between the cultivars tested. The Vapro and WP4C provide greater applicability than the conventional P–V method to studies requiring both high throughput and high sensitivity. Consistency of measurement type is however highly recommended in future studies as some differences were observed between Vapro and WP4C.

**Electronic supplementary material:**

The online version of this article (10.1186/s13007-019-0410-3) contains supplementary material, which is available to authorized users.

## Background

Trees within the urban environment often experience abiotic stresses [1–3]; that in-turn, can increase susceptibility to pest and diseases [4]. Selecting for drought tolerance in urban tree species can have a significant influence on survival rates, aftercare requirements and future aesthetic and environmental benefits [5–7]. Tree selection is often focused on aesthetic characteristics [8], however, when tolerance is considered it is often based on personal experience and observation. Data from plant-use literature and scientific studies is frequently inconsistent between sources and often lacks specificity [5, 9]. Increases in the frequency and severity of drought events are expected as a result of climate change [10–12]. Informed tree selection based on physiological or genetic drought tolerance traits is therefore increasingly desirable, facilitating selection for current and future environmental demands [13]. Foliar physiological traits are gaining popularity as they can determine physiological drought tolerance as opposed to drought avoidance strategies [5]. Genotypes which avoid drought may shed leaves or branches in response to drought stress or rely on extensive root systems to gather water [14, 15], these strategies are not desirable for urban sites. Urban tree selection is clearly more nuanced than simply consideration of functional traits; however, improvements to current tolerance information is essential to aid and encourage appropriate selection [16]. One physiological trait capable of identifying drought tolerance is the measurement of leaf water potential at wilting or turgor loss (π_tlp_) [17, 18]. This trait is capable of characterising intraspecific drought tolerance [5]. Techniques are now available to increase the speed of this measurement [19] facilitating ecological scale studies [20] and studies to aid appropriate tree selection between and within genera [5, 9, 21]. Therefore, a range of approaches are currently being used to determine π_tlp_. However, no study has evaluated the sensitivity of these alternative methods among closely related cultivars. In this study, the so-called direct measurements, using a vapour-pressure osmometer and dewpoint hygrometer to measure water potential are compared with a classical pressure–volume (P–V) curve method, measured on adjacent leaves. In this study, closely related genotypes are used to allow the sensitivity of measurement method to be evaluated.

P–V curves are the classical method of inferring a range of plant-water relation parameters [22] and can provide information on genotypic drought tolerance using the parameter π_tlp_ [18, 23, 24]. A more negative π_tlp_ lengthens the functional range of foliar water potential [25] and is thought to be achieved by a combination of osmotic adjustment (solute accumulation to increase cell hydration) and elastic adjustment (decreasing the point at which turgor loss occurs) [26, 27]. π_tlp_ is now considered the dominant determining factor of drought tolerance [18, 27]. The production of P–V curves has one significant disadvantage; they are time-consuming to produce, meaning adequately large scale studies and genetic screening are impractical [26, 28, 29]. Additionally, despite P–V curves being widely regarded as the classical method for determining water relation parameters, the comparative accuracy between methods has been frequently criticised [30, 31]. Warranting further studies investigating alternative methods of measuring water potential (Ѱ) in plant tissue [28, 32, 33].

Direct measurements (vapour-pressure osmometer and dewpoint hygrometer), are rapid methods used to determine water potential [28]. The dewpoint hygrometer (such as the WP4C, decagon devices Inc. München, Germany) uses the chilled-mirror dewpoint technique [34] measuring water potential from zero to − 300 MPa on ca. 35 mm diameter leaf discs [35, 36]. The dewpoint hygrometer measures the sum of osmotic and matric potential; it has been used successfully on leaves of tobacco and ivy [35] and flowers of slipper orchid [37]. Relative difference between dewpoint hygrometer and pressure chamber measurements of water potential have been shown to be very similar (R^2^ = 0.84) [33]. The vapour-pressure osmometer (Vapro^®^, Wescor, Logan UT, USA) measures solute concentration (osmolality) which can be converted to water potential using the Van’t Hoff equation (Eq. 1); it can measure leaf discs of ca. 8 mm diameter [20, 28] or expressed sap [28, 38]. Callister et al. [28] show osmometer osmotic potential (π) measurements of expressed sap are comparable with those of parallel π pressure chamber readings. Bartlett et al. [19] show that measurements of π on rehydrated freeze-thawed leaf discs can rapidly determine the osmotic potential at full rehydration or full turgor (π_0_). Bartlett et al. [19] also demonstrated that π_0_ correlates to the π_tlp_. They used vapour-pressure osmometer measurements taken from plants which had pressure chamber derived P–V curves, determined within 4 weeks of each other, for sixteen species. However, for fourteen additional species, the P–V curves had been calculated within the previous 2 years [19]. Significant adjustment of π_tlp_ is known to occur across a single season [5]; however, additional meta-analysis has also shown a good correlation between π_0_ and π_tlp_ [18], adding further validity to the measurement despite potential issue with the timing of the initial data collection. Sufficient evidence now exists to warrant large scale evaluations of π_tlp_, calculated from π_0_, using a vapour-pressure osmometer [5, 9, 20, 21]. However, it is not yet clear if a dewpoint hygrometer can be used to evaluate π_tlp_. Therefore, this study aims to evaluate the accuracy of osmometer and hygrometer measurements in direct parallel to P–V curves using very closely related *Acer* genotypes.

## Method

### Plant material

Thirty-two seven-year-old, 4 m tall trees were used for this experiment arranged across three completely randomized linear rows. The following *Acer* genotypes were measured during this trial: *A. platanoides* ‘Drummondii’, *A. p.* ‘Emerald Queen’, *A. p.* ‘Royal Red’, *A. p.* ‘Princeton Gold’ and *A. pseudoplatanus* ‘Negenia’. All measured cultivars were grafted onto their respective species-type rootstocks. Trees were potted during the winter of 2013/14 and grown at Barcham Trees nursery, Ely, Cambridgeshire, UK (52.366923°N, 0.315864°W) prior to being planted outside in March 2017 at the Bartlett Tree Research laboratory, Shinfield, Reading, Berkshire, UK (51.412393°N, − 0.937909°W). Encircling roots were cut on all trees to aid establishment during the planting process. Trees were arranged across three rows, each measured cultivar was randomized within each row.

### Sample preparation

Two visually healthy leaves were removed ca. 30 cm below a terminal bud on the lower limb (ca. 2 m high) of each tree; opposite leaves were selected to ensure the closest similarity in physiological age. Leaves were collected between 16:00 and 17:00 on the 24th July to the 9th of August 2017. Leaves were removed from the tree by snapping at the axil union and immediately returned to the laboratory (within < 2 min). In the laboratory, leaves were immediately weighed and petioles re-cut underwater (ca. 1 cm away from the petiole base), petioles and cut petiole portions were left in water to fully hydrate in the dark for ca. 12 h. Hydrating leaves were left in an insulated container during this time kept near 100% relative humidity [average vapour-pressure deficit [39] equalled 0.01 (± 0.03)]. Individual, fully hydrated leaves were removed from the container, patted dry and immediately weighted and processed using either the pressure chamber P–V curve method or direct methods.

### Pressure–volume curves

**Fig. 1:**
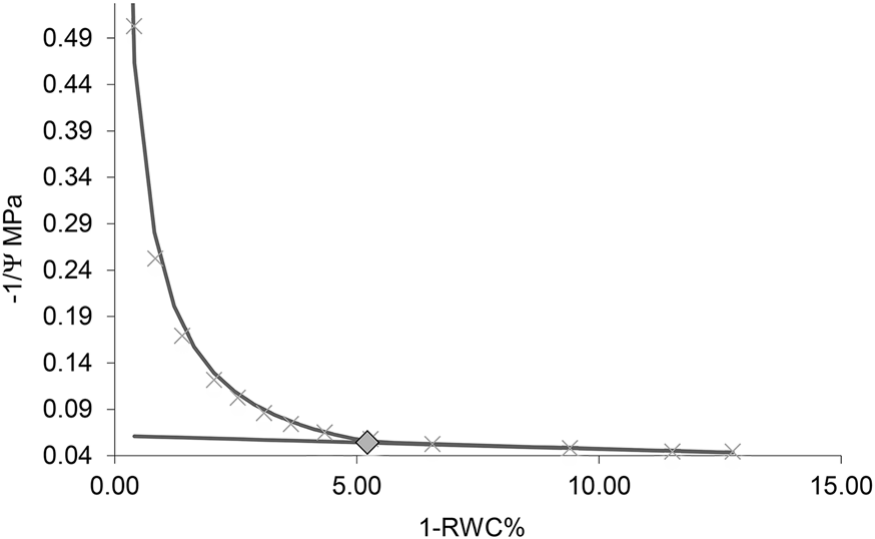
Representative pressure–volume curve. The square indicates the turgor loss point

Pressure–volume curves were calculated in accordance with the sap expression method; the method was similar to that used by Parker and Pallardy [31]. Whole undamaged leaves were sealed inside a pressure chamber (model 600D, PMS instruments Co., Albany, USA) with a piece of damp filter paper to reduce water loss. The average initial balance pressure was − 0.13 MPa (± 0.007). Leaves which did not hydrate to an initial Ѱ of > − 0.2 MPa were discarded [25]. Incremental pressures of 0.2 MPa were applied to the leaf, beginning at 0.2 MPa. P–V curves were halted at − 2.4 MPa or when greater than three data points were in the linear portion of the graph. Total expressed sap at each pressure was absorbed in pre-weighed 1.5 ml Eppendorf tubes filled with dry low-lint absorbent tissue paper (Kimtech Science, Kent, UK). Tubes were handled and opened for the minimum possible time during sap collection to prevent evaporation. Leaves were weighed immediately following the final measurement, facilitating determination of the average uncollected water (4.7%). Leaves were then dried for > 48 h at 60 °C. P–V curves were plotted as 100-RWC (relative water content) (D) on the x axis, against − 1/MPa (y axis). Overhydration, or plateau effects were corrected where appropriate in accordance with the method described by [40]. Water potential at the turgor loss point (π_tlp_) was calculated based on a method developed by Schulte and Hickley [41], obtained from: landflux.org/resources/PV_Curve_Fitting_5.6.xls. This method has also been used by [42–44] (Fig. 1).

### Direct measurements

Two leaf discs, 35 mm and 8 mm diameter, (dewpoint hygrometer and vapor pressure osmometer respectively) were taken between the mid-rib and margin on the lower quartile of the opposing leaf used in the P–V curve. Leaf discs were foil wrapped and submerged in liquid nitrogen. Prior to the measurement, leaf discs were punctured 10–15 times with sharp-tipped forceps to improve equilibration times [19].

Dewpoint hygrometer (WP4C, decagon devices Inc. München, Germany) measurements were taken with the device in its continuous mode, connected to the AquaLink data logging software (decagon devices) on a laptop computer. One measurement per leaf was recorded when values became stable (ca. 15–20 min.). Stability was assessed graphically for each leaf disc. The WP4C measures total water potential which is the sum total of gravitational, matric, osmotic and pressure potentials. In freeze thawed leaf discs it is putatively assumed that gravitational, matric and pressure potentials are all zero or negligible, therefore, in this study, osmotic potential is the considered component.

Osmometer measurements were taken with a vapour pressure osmometer (Vapro 5600, Wescor, Logan UT, USA) using the standard 10 µl chamber. Measurements were made in accordance with the method detailed by Sjöman et al. [9].

For measurements made using the Osmometer, solute concentration (mmol kg^−1^) was converted to water potential using Van ‘t Hoff’s equation:

1 $$\pi_{0} = - CRT$$

Equation 1 Van ‘t Hoff equation, where C is the molar solute concentration (mmol kg^−1^), R is the universal gas constant (8.3144598E−0.6) in m^3^ MPa K^−1^ mol^−1^, T is the temperature (K) [45].

Dewpoint hygrometer and vapor pressure osmometer are hereafter referred to as WP4C and Vapro for simplicity.

Both direct measurements of osmotic potential (π) were converted into predicted P–V value ($\hat{\pi }_{pv}$) using the equation determined by Bartlett et al. [19] (Eq. 2).

2 $$\hat{\pi }_{pv} = 0.587\pi - 0.546$$

Equation 2 conversion from osmometer measurement (π) to predicted P–V ($\hat{\pi }_{pv}$) measurement [19].

π_tlp_ was calculated from π_0_ using the regression equation adapted for temperate species by Sjöman et al. [5] originally calculated from Additional file 1 published by [18].

3 $$\uppi_{\text{tlp}} = - 0.2554 + 1.1243 \times\uppi_{0}$$

Equation 3 Adapted equation facilitating prediction of π_tlp_ (Ѱ_P0_) from osmometer π_0_ (Ѱ_π100_) (R^2^ = 0.91) (notation in parentheses is the notation used by Sjöman et al. [5]. The notation used here correspond to Bartlett et al. [18].

### Statistical analysis

Statistical analysis was performed using GenStat 17th edition. Following tests for normality, analysis of variance (ANOVA) was used to test for differences between means. Linear regression (R^2^) and Spearman’s rank correlation coefficient (r_s_) was also calculated in order to describe the relationship between readings. Post-hoc analysis was performed using a Tukey’s 95% confidence interval.

## Results

A significant effect of both genotype and measurement method (p ≤ 0.001 for both) was observed. However, a significant interaction between genotype (cultivar) and method was observed following a two-way ANOVA (p ≤ 0.001). Data was therefore compared overall with cultivars nested within measurement method.

Similarities between measurements was determined using a correlation coefficient (R^2^) and Spearman’s rank correlation coefficient (R_s_). P–V measurements were excluded from correlation comparisons as no significant differences were discovered between cultivars. The Vapro and WP4C provided the same rank (R_s_ = 1) and highly similar correlation coefficient (R^2^ = 0.96). Values of π_0_ provided the same comparative ranking as π_tlp_.

**Fig. 2:**
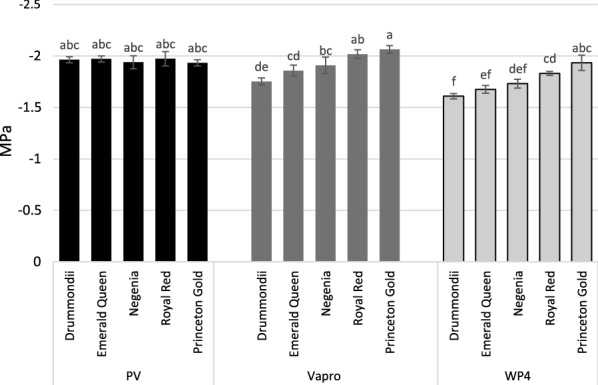
Showing π_tlp_ for each method, Vapro and WP4 are reported as π_tlp_ (P–V) following conversion using Eq. 2. Error bars show standard error. Letters denote significant differences (Duncan multiple range test) between cultivars nested in method at the 95% confidence interval. A. platanoides ‘Drummondii’, A. p. ‘Emerald Queen’, A. p. ‘Royal Red’, A. p. ‘Princeton Gold’ and A. pseudoplatanus ‘Negenia’. Between species analysis for each measurement method p ≤ 0.001 for Vapro and WP4C, p = 0.938 for P–V π_tlp_

Correcting measurements using Eq. 2 is highly important, especially if values are to be compared against P–V curve data. Equation 2 improved similarity to P–V curves by an average of ca. 5% for both Vapro and WP4C. However, in this study significant and species-specific differences occurred with both devices when compared to the P–V method (Fig. 2).

## Discussion

In this study, the pressure chamber pressure–volume (P–V) curve method was unable to identify significant differences between the closely related cultivars tested (p = 0.938). However, both direct measurements tested (WP4C and Vapro), identified highly significant differences between cultivars (p ≤ 0.001). This is the first study to our knowledge to demonstrate the suitability of the dewpoint hygrometer (WP4C) in comparison to the P–V curve and vapour-pressure osmometer (Vapro) methods when measuring π_tlp_. Significant differences between measurement methods were present for all cultivars except *A. pseudoplatanus* ‘Negenia’ (p = 0.092) and *A. platanoides* ‘Princeton Gold’ (p = 0.112) despite the use of the correction factor described by Bartlett et al. [19] (Eq. 3). No difference in rank however was observed between the WP4C and Vapro (R_s_ = 1). The Vapro returned results comparably closer to those from P–V curves. The Vapro and WP4C differed from P–V values at an average 0.04 MPa (± 0.055) and 0.20 MPa (± 0.057) respectively, these differences are not however thought to be practically significant for species selection. Therefore, either device can be utilised for tolerance studies.

As suggested by Zhang et al. [37] and Martínez et al. [33] more negative values (average − 26.5%, without correction, Eq. 3) were observed using both devices in comparison to the pressure chamber. Many theories exist to explain why thermocouple and hygrometer devices measure more negatively than pressure chambers, including water loss during leaf excision as well as active accumulation of solutes by neighbouring undamaged tissue [33, 46]. Zhang et al. [37] however, also discuss simply that the measurement is of the air above the sample, thus a more negative Ѱ is returned. It is however imperative that the air above the sample is in equilibration with the sample, consequently we assume Zhang et al. [37] discussion is based on the assumption that losses in water potential may occur in locations where sample water potential is more negative than ambient humidity. Therefore, a decrease in sample water potential would occur in order to reach equilibration. If this was the case, more negative values would be expected from the WP4C owing to the greater leaf to chamber volume (0.27 mm^3^ ml^−1^ vs 2.3 mm^3^ ml^−1^ for WP4C and Vapro respectively); in this trial this did not occur.

In some circumstances, utilisation of the Vapro device can be recommended; the larger leaf disc size required by the WP4C, reduces the ability to evaluate plants with smaller or more complex leaf areas without adaptation of the method. Previous studies have also utilised the Vapro to evaluate relatively large genotypic selection [5, 9, 21]. In future studies, we recommend a process of cross calibration with previous studies using species in common in order to place genotypes within the drought tolerance continuum.

## Conclusion

The Vapro and WP4C provide greater applicability than the conventional P–V method to studies requiring high throughput and high sensitivity. Data presented here reveals the sensitivity of the vapour-pressure osmometer and dewpoint hygrometer methods to measure π_tlp_ characterising the drought tolerance of closely related genotypes. Data identifies no difference in rank between results from both WP4C and Vapro. Some significant differences were however observed between Vapro and WP4C (Fig. 2) therefore consistency of measurement type is recommended in future studies. Poor sensitivity was observed when using the P–V method, therefore, future studies should utilise either the vapour-pressure osmometer or dewpoint hygrometer in order to provide rapid and sensitive genotypic drought tolerance quantifications.

## Additional file

**Additional file 1.**All data, P-V curves raw data, WP4, Vapro, & P-V TLP values.

## Abbreviations

π_tlp_: water potential at turgor loss point
π: osmotic potential
π_0_: osmotic potential at full rehydration or full turgor
Ψ: water potential
R_s_: Spearman’s rank correlation coefficient
P–V: pressure volume
$\hat{\pi }_{pv}$: predicted pressure–volume osmotic potential
